# An Insight Based on Computational Analysis of the Interaction between the Receptor-Binding Domain of the Omicron Variants and Human Angiotensin-Converting Enzyme 2

**DOI:** 10.3390/biology11050797

**Published:** 2022-05-23

**Authors:** Ismail Celik, Magda H. Abdellattif, Trina Ekawati Tallei

**Affiliations:** 1 Department of Pharmaceutical Chemistry, Faculty of Pharmacy, Erciyes University, Kayseri 38039, Turkey; ismailcelik@erciyes.edu.tr; 2 Department of Chemistry, College of Science, Taif University, P.O. Box 11099, Taif 21944, Saudi Arabia; m.hasan@tu.edu.sa; 3 Department of Biology, Faculty of Mathematics and Natural Sciences, Sam Ratulangi University, Manado 95115, North Sulawesi, Indonesia

**Keywords:** Omicron, spike protein, receptor-binding domain, binding mechanism, computational analysis

## Abstract

**Simple Summary:**

The Omicron variant has recently been divided into BA.1, BA.2, and BA.3 subvariants. In the present study, we focused on comparing the interaction between the receptor-binding domain (RBD) of BA.1 and BA.2 spike proteins with human angiotensin-converting enzyme 2 (hACE2) using a computational approach. The RBD BA.2 was modeled after the BA.1. The results from molecular docking and molecular dynamics studies showed that RBD BA.2 has a higher and more stable affinity for hACE2 compared to RBD BA.1.

**Abstract:**

Concerns have been raised about the high number of mutations in the spike protein of the new emergence of the highly transmissible Omicron variant (B.1.1529 lineage) of severe acute respiratory syndrome coronavirus 2 (SARS-CoV-2). This variant’s extraordinary ability to evade antibodies would significantly impair the current vaccination program. This present study aimed to computationally analyze the interaction between the receptor-binding domain (RBD) in the spike protein of Omicron variants and human angiotensin-converting enzyme 2 (hACE2). The docking results indicated that Omicron BA.2 has exceptionally strong interactions with hACE2 in comparison to Omicron BA.1, Delta, and wild-type, as indicated by various parameters such as salt bridge, hydrogen bond, and non-bonded interactions. The results of the molecular dynamics simulation study corroborate these findings, indicating that Omicron BA.2 has a strong and stable interaction with hACE2. This study provides insight into the development of an effective intervention against this variant.

## 1. Introduction

**Figure 1:**
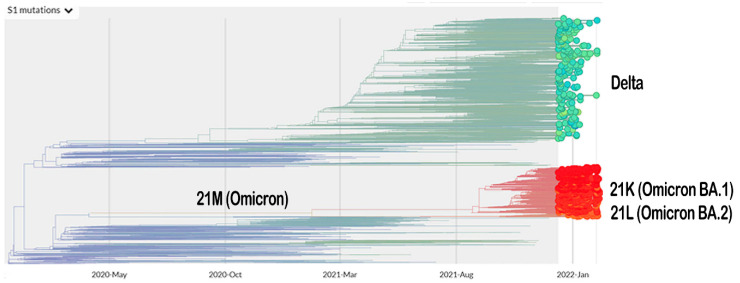
The phylogeny of Omicron and Delta variant based on mutation on their Spike protein (https://nextstrain.org/ncov/gisaid/global?c=S1_mutations&dmin=2021-12-13; accessed on 11 February 2022).

The vaccination program for coronavirus disease 2019 (COVID-19) is currently underway. Meanwhile, the severe acute respiratory syndrome coronavirus 2 (SARS-CoV-2), which is responsible for the COVID-19 pandemic, is continuing to mutate. The Omicron variant (B.1.1.529 lineage, clade 20 K), the new emerging variant of this virus, has elicited panic responses worldwide due to its contagious nature and ability to evade vaccine-induced immunity and therapeutic monoclonal antibodies [1,2,3,4]. The majority of Omicron mutations occur on the spike protein, altering the binding epitopes of many currently available antibodies [5]. The phylogeny of Omicron, based on Spike-protein mutation related to the Delta variant, is presented in Figure 1.

The Omicron variant outperforms the Delta variant when it comes to evading vaccinated individuals’ immunity. Delta had a higher positivity rate (5.25%) than the Omicron variant (4.5%) among unvaccinated and single-vaccine-dose individuals. On the other hand, Omicron had a higher rate of positivity than Delta in those who received two or three vaccination doses [1]. However, the booster dose of mRNA-based vaccines significantly reduces the risk of a person contracting SARS-CoV-2 and becoming ill [6,7,8,9]. 

Recently, Omicron was subdivided into three lineages (BA.1, BA.2, and BA.3) based on their unique mutations [10]. Both BA.1 and BA.3 have the 69–70 deletion on the spike, which is not detected in BA.2, thus making it an outlier lineage. These deletions cause a negative signal when certain polymerase-chain reaction (PCR) assays are used to detect the S gene target [11]. According to one study, there are no specific mutations in the spike protein that are unique to the BA.3 lineage. Rather than that, it is the result of BA.1 and BA.2 spike protein mutations. [10]. Common mutations in ORF1ab of the BA.1 and BA.2 lineages are T3255I, P3395H, SGF3675del, P4715L, and I5967V. The BA.1 lineage contains five mutations (K856R, SL2083I, A2710T, L3674F, and I3758V) that are unique to it. On the other hand, the BA.2 lineage has unique mutations at S135R, T842I, G1307S, L3027F, T3090I, L3201F, F3677L, R5716C, and T6564I [12]. Omicron was suggested to have evolved from clade 20B rather than Delta [13]. The 21 most common mutations detected in the spike protein of the three lineages are G142D, G339D, S373P, S375F, K417N, N440K, S477N, T478K, E484A, Q493R, Q498R, N501Y, Y505H, D614G, H655Y, N679K, P681H, N764K, D796Y, Q954H, and N969K. The Q498R and N501Y mutations are believed to contribute to the increased binding of the virus to the hACE2 receptor, whereas the H655Y, N679K, and P681H are thought to increase the cleavage of the spike and facilitate the transmission of the virus [10]. The E484A in Omicron (and E484K for Beta and Gamma) are involved in immune escape [14]. 

Omicron has a high frequency of mutations in its spike protein [14], which is the ultimate target for neutralizing antibodies [15]. Due to these mutations, which most likely result in a change in the conformation of the spike protein [16], it is possible that the viral biology will be altered. This could include changes in pathogenicity, infectivity, transmissibility, and/or antigenicity, as well as alterations in antibodies’ ability to recognize and neutralize the virus [17]. The receptor-binding domain (RBD) is a component of the spike protein that is essential for viral entry into the cells [18]. RBD specifically identifies the host angiotensin-converting enzyme 2 (ACE2) as its receptor [19,20], allowing the virus to enter the cell via direct fusion of the viral envelope with the host’s cell membrane, or via endosomal membrane fusion following endocytosis [21].

An in silico analysis revealed that mutations in RBD had a significant effect on the spike (S) protein’s structural behavior when compared to the wild type (WT) of SARS-CoV-2, which also had an effect on RBD’s binding to human ACE2 (hACE2) at the respective sites [22]. This may contribute to the high affinity of the virus for hACE2, resulting in increased transmissibility [23,24]. To better understand the interaction between RBD of Omicron variants and hACE2, in the present study, we conducted a computational analysis using molecular docking and molecular dynamics simulation. Additionally, the affinity of RBD WT and Delta for hACE2 was also investigated. Our finding revealed that Omicron BA.2 had a stronger and more stable interaction with hACE2 compared to other variants under study, implying its higher affinity for hACE2.

## 2. Materials and Methods

### 2.1. Multiple Sequence Alignments

The three-dimensional (3D) structures of the RBD of SARS-CoV-2 Omicron BA.1 and Delta that bind to hACE2 were retrieved from the Protein Data Bank (PDB) with the PDB identifiers (IDs) 7WBP and 7WBQ [25], respectively. The Omicron BA.2 was modeled based on the BA.1 sequence, using an in silico mutagenesis approach [26]. The sequences were aligned using the UCSF Chimera package release 1.16 [27].

### 2.2. Homology Modeling

The modeling of the spike protein RBD unit of the omicron BA.2 subvariant was performed via the SWISS-MODEL web server [28]. The quality of the protein structure was validated in ProSA-web [29]. The PROCHECK server [30] was used to determine the accuracy of the protein model, which was then visualized in a Ramachandran plot [31]. A define secondary structure of proteins (DSSP) algorithm [32] was employed to assign the secondary structure of the modeled protein.

### 2.3. Molecular Docking Study

The 3D structure of the RBD Omicron BA.2 were minimized on the SWISS-MODEL web server [28]. The protein–protein docking simulations were carried out using the HDOCK server [33]. For molecular docking, validation, and comparison of interaction energies, hACE2 redocking was performed with Omicron BA.1 and Delta RBD units, respectively. The Prodigy web server [34] was used to carry out the binding affinity (ΔG) calculation and dissociation constant (Kd) analysis of the complex. The PDBsum web server [35] was utilized for the visualization of the interaction network, which included salt bridges, hydrogen bonds, and non-bonded contacts. The 3D interaction was visualized in PyMOL Molecular Graphics System version 2.4.1.

### 2.4. Molecular Dynamics Simulations Study

The molecular dynamics simulations (MDS) study was conducted in accordance with the previously described procedures [22]. The simulation was carried out in Gromacs 2020.4 [36] in order to investigate the dynamic behavior and binding interaction of the RBD-hACE2 complexes. Input files required for MDS were created with the CHARMM-GUI [37] server. The topology of the RBD-hACE2 complexes was built using CHARMM36m force fields [38] and the TIP3 water model [39]. A rectangular box applying 3D periodic boundary conditions was selected for solvation at 15 Å distance between the edges of the box and the protein–protein complex [40]. The system was neutralized with 0.15 M KCl by the Monte Carlo method. The steepest descent integrator was used to perform the energy minimization in 5000 steps. Equilibration of the system was performed using 0.5 ns NVT/NPT ensembles at 303.15 K and 1 atm pressure using the Nose–Hoover thermostat [41] and Parrinello–Rahman barostat [42], respectively. The leap-frog integration method was used to perform 100 ns molecular dynamics (MD) simulations of 1000 frames in time steps of 2 fs. The MD trajectory analysis was carried out using the scripts gms rms, rmsf, and gyrate for the root-mean-square deviation (RMSD), root-mean-square fluctuation (RMSF), and the radius of gyration (Rg), respectively. The graphs were created using QtGrace version 0.2.6. The MD trajectory videos were created with the PyMOL Molecular Graphics System version 2.4.1.

## 3. Results

### 3.1. Multiple Sequence Alignments Result

**Figure 2:**
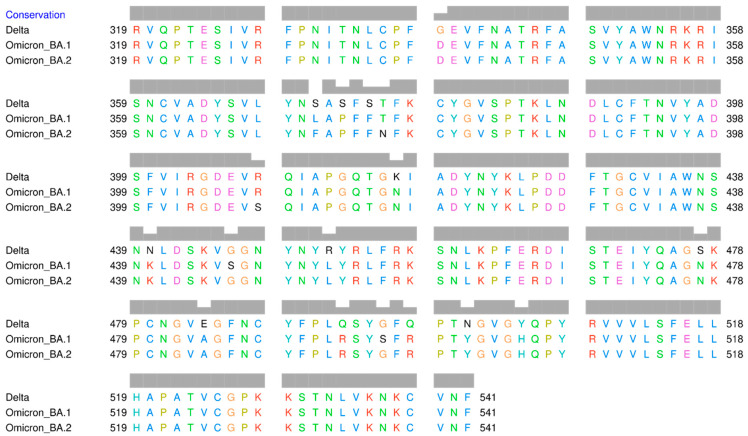
Multiple sequence alignment of the receptor-binding domain of SARS-CoV-2 Delta and Omicron variants.

The result of multiple sequence alignments (MSA) of the RBD amino acid sequences of Omicron BA.1, Omicron BA.2, and Delta variants is displayed in Figure 2. The mutation sites that have been identified are listed in Table 1. There are 15 amino acid mutations in RBD that distinguish the three variants. Additionally, there are several positions where the same amino acid mutation occurs between Delta and Omicron BA.1, Delta and Omicron BA.2, as well as between Omicron itself. At position 371, all amino acids are different, with serine (S) in Delta, leucine (L) in Omicron BA.1, and phenylalanine (F) in Omicron BA.2. Serine and leucine are both neutral and polar amino acids, whereas phenylalanine is non-polar.

Numerous mutation sites exhibit changes in the polarity of amino acids, such as at position 373, where serine (polar) in Delta, but proline (non-polar) in Omicron. At position 446, glycine (G) (non-polar) is present in Delta and Omicron BA.2, but serine (polar) is present in Omicron BA.1. At position 452, arginine (R) (polar) in Delta, but leucine (L) (non-polar) in both Omicron variants. At position 484, Delta has the polar glutamic acid (E), whereas both Omicron variants have the non-polar alanine (A). At position 496, Delta has non-polar glycine (G), whereas both Omicron variants have polar serine.

### 3.2. Homology Modeling of RBD of Omicron BA.2 Variant

**Figure 3:**
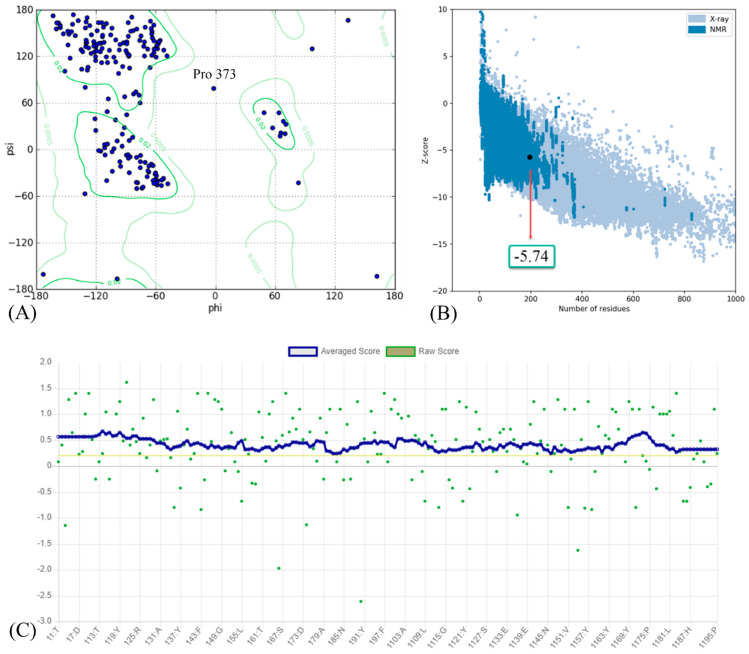
(**A**) Ramachandran plot; (**B**) Z-score; and (**C**) residue scores plot for RBD of Omicron BA.2 variant.

The results of the homology modeling assessment of the Omicron BA.2 variant are presented in Figure 3. The Z-score for Omicron BA.2 generated by the ProSA web server was −5.74. The negative value indicates the validity of the protein modeling result. Additionally, the model meets the requirements because its most favored region in the Ramachandran plot is greater than 90%. A DSSP model for secondary structure assignment showed that 100% of the residues had an average 3D/1D score of ±0.2. The structure is considered valid if at least 80% of the amino acids in the 3D/1D profile have a score of ±0.2.

### 3.3. Protein–Protein Interaction Revealed by Molecular Docking Study

The interaction of RBD and hACE2 was investigated using a molecular docking approach on the HDOCK server. The best model was chosen for further analysis. The predicted HDOCK score, ligand RMSD, ΔG and Kd calculations of the protein–protein complex are presented in Table 2. The Kd values of the complexes Delta-ACE2 and Omicron BA.2-ACE2 were similar, with a value of 1.5 × 10^−9^ M and 1.4 × 10^−9^ M, respectively. Both had lower Kd values compared to Omicron BA.1-ACE2 (1.5 × 10^−8^ M). This value seems to correspond to the binding affinity value, where both Delta-ACE2 and Omicron BA.2-ACE2 complexes had ΔG values of −12.5 and −12.6 kcal/mol, respectively. Meanwhile, the Omicron BA.1-ACE2 complex yielded −11.1 kcal/mol. The HDCOK score supported the finding that Omicron BA.1-ACE2 had the most negative value, indicating this complex had the highest binding affinity.

**Figure 4:**
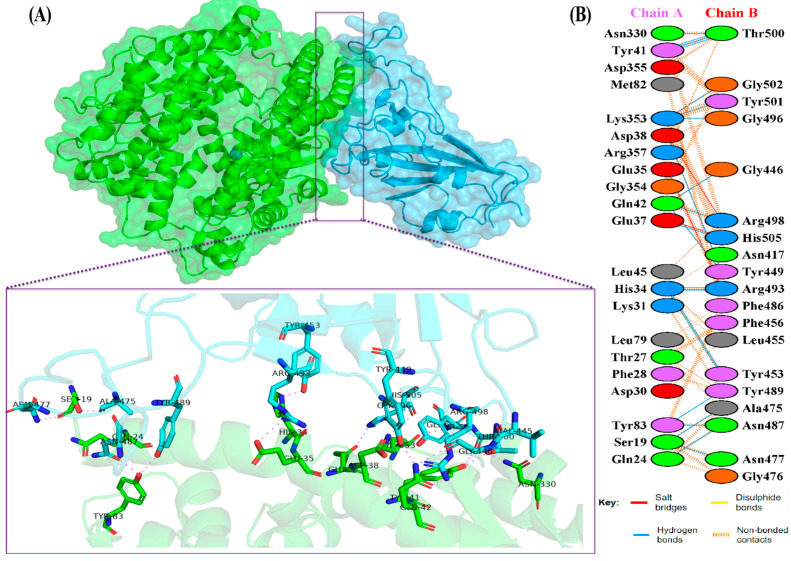
(**A**) The 3D visualization of Omicron BA.2-ACE2 complex interaction (ACE2 is denoted in green, whereas BA.2 RBD is denoted in blue); (**B**) interface of Omicron BA.2-ACE2 complex interaction. Residue colors: positively charged (blue): His, Lys, Arg; negatively-charged (red): Asp, Glu; neutral (green): Ser, Thr, Asn, Gln; aliphatic (gray): Ala, Val, Leu, Ile, Met; aromatic (purple): Phe, Tyr, Trp; Pro; and Gly (orange).

**Figure 5:**
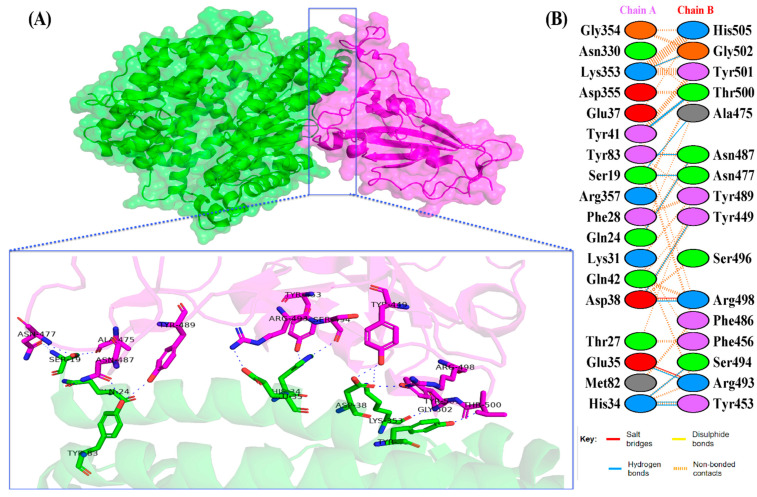
(**A**) The 3D visualization of Omicron BA.1-ACE2 complex interaction (ACE2 is denoted in green, whereas BA.1 RBD is denoted in magenta); (**B**) interface of Omicron BA.1-ACE2 complex interaction. Residue colors: positively charged (blue): His, Lys, Arg; negatively charged (red): Asp, Glu; neutral (green): Ser, Thr, Asn, Gln; aliphatic (gray): Ala, Val, Leu, Ile, Met; aromatic (purple): Phe, Tyr, Trp; Pro; and Gly (orange).

The docking results of the Omicron-hACE2 complexes are presented in Figure 4 and Figure 5. The interface statistics and the type of interaction that was analyzed using the PDBsum webserver are presented in Table 3 and Table 4, respectively. The results were compared with our previous results on WT [22]. Omicron B.2. had the highest number of interface residues of non-bonded contacts, hydrogen bonds (H-bonds), and salt bridges. The RBD and hACE2 complexes of SARS-CoV-2 variants differ greatly in their amino-acid–amino acid interactions. The most striking difference between Omicron, Delta, and WT is in the salt bridges, where Asp30 in hACE2 interacts with Lys417 in Delta and WT. Meanwhile, salt bridges on Omicron occur between Glu35 and Asp38 with Arg493 and Arg498 in BA.1 and BA.2, respectively. In all of the hACE2-RBD variants and WT, the H-bonds at Gln24-Asn487, Tyr41-Thr500, Tyr83-Asn487, and Lys353-Gly502 were found. The H-bonds in both Omicron variants were found at Ser19-Asn477, Ser-Ala475, Gln24-Asn487, His34-Tyr453, Asp38-Tyr449, Tyr41-Thr500, Tyr83-Asn487, and Lys353-Gly502. Salt bridges were found at Glu437-Arg493 and Asp48-Arg498 in both Omicron variants. The addition of H-bonds to His34-Arg493, Tyr41-Thr500, Gln42-Arg498, Tyr83-Tyr489, Asn330-Thr500, and Lys353-Tyr501, as well as the salt bridge Glu-37-His505, appears to make Omicron BA.2 more infective than Omicron BA.1, Delta, and WT.

### 3.4. Molecular Dynamics Simulations Results

**Figure 6:**
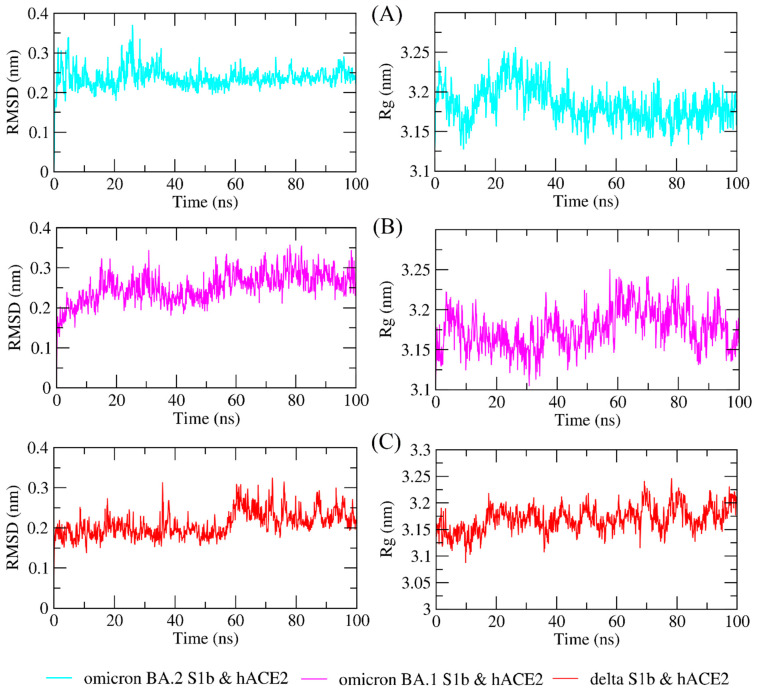
The molecular dynamics simulation trajectory analysis of protein–protein complexes. Dynamic behavior and stability of the interaction between hACE2 and (**A**) Omicron BA.2, (**B**) Omicron BA.1, and (**C**) Delta variants’ RBDs based on the root-mean-square deviation (RMSD) and the radius of gyration (Rg).

**Figure 7:**
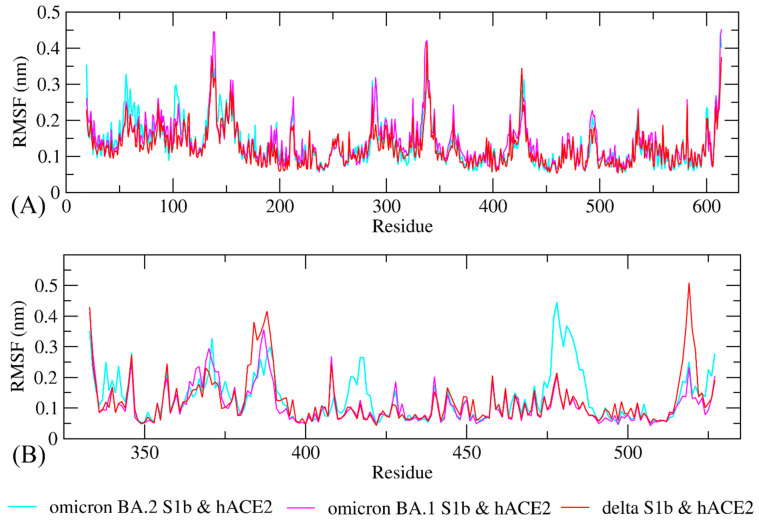
Conformational change and flexibility for each residue of the protein–protein complex of the RBD of Omicron BA.2, Omicron BA.1, and Delta variants with hACE2. (**A**) Root-mean-square fluctuation (RMSF) plot of hACE2s and (**B**) RMSF plot of Omicron BA2, BA1, and Delta variants RBD.

The study of molecular dynamics simulations is extremely beneficial in gaining a thorough understanding of how molecules interact. Figure 6 and Figure 7 depict the dynamic behavior and stability of the interaction between hACE2 and variant RBDs. Supplementary Materials contains videos demonstrating the MDS of all complexes. The average RMSD trajectory value of all complexes remained steady, below 0.4 nm, for the whole of the simulation process. In comparison to the hACE2-Omicron BA.1 and hACE2-Delta complexes, the hACE2-Omicron BA.2 complex has a more stable RMSD profile after 40 ns to the end of the simulation (100 nm) and fluctuated at 0.2–0.3 nm. The RMSF profiles of all complexes were similar, and the amino acid average deviation was below 0.5 nm, although fluctuations appeared to range between 0.2 and 0.3 nm. However, BA.2 showed more flexibility at the residue 475 to 485. The levels of compaction were reflected by Rg. The Rg values fluctuation observed during simulations of all systems was attributed to the complex’s binding and dissociation. The Rg plot showed that in all variants, the compactness of the protein fluctuated below 3.25 nm. Omicron BA2, on the other hand, appeared to be stable from 40 ns to the end of the simulation, fluctuating between 3.15 and 3.20 nm. During the simulation, the fluctuation range of Rg from Omicron BA.1 and Delta was greater, ranging between 3.1 and 3.25 nm. Hence, based on this Rg value, it appears that BA.2 is more stable than the other two variants.

## 4. Discussion

SARS-CoV-2 continues to mutate, giving rise to numerous variants. At present, the Omicron is the most contagious variant. Most mutations of this variant occur in the Spike protein, particularly in the N-terminal domain (NTD) and receptor-binding domain (RBD). The Spike protein is the most important factor in the virus’ ability to infiltrate the host cell. The virus enters cells via a variety of mechanisms, one of which is the attachment of the Spike protein to hACE2. Mutations in the Spike protein, specifically in the receptor-binding domain, appear to affect viral attachment to hACE2. As a result, the present study investigated the consequence of RBD mutations in the Omicron variant on its binding affinity to hACE2.

The predicted Z-score of the modeled Omicron BA.1 protein indicates that the modeling result is satisfactory, implying that there are only a few incorrect structures and thus a high degree of similarity to the native protein. The score fell within the normal range for proteins of comparable size [43], indicating that the modeling produced a highly reliable structure. The Ramachandran plot analysis and the DSSP model validate the results of successful modeling. A high-quality model should have a coverage rate of at least 90% in the most favored regions [44,45].

Molecular docking analysis was used to decipher protein–protein interactions. The results of the analysis, which included HDOCK energy score, binding affinity, dissociation constant, the number of interface interactions, H-bonds, salt bridge, and non-bonded (electrostatic and van der Waals) interactions, all indicated that Omicron BA.2 interacts best with hACE2 when compared to Omicron BA.1 and Delta variants. The similar finding was shown by the result of previous study that the binding-free energy (BFE) for WT-RBD-ACE2 complex was −37.44 (kcal/mol), while BA.3, BA.2, and BA.1 subvariants had a BFE of −73.55, −72.36, and −70.6 kcal/mol, respectively when assessed using HawDock server [46]. The HDOCK energy score is comparable to the dampened molecular mechanics Poisson–Boltzmann surface area (dMM-PBSA) value [47]. Protein folding is primarily driven by the hydrophobic effect. However, electrostatic interactions also contribute significantly to the folding, stability, flexibility, and function of proteins [48]. Meanwhile, van der Waals interactions play a role in the stability and function of proteins [49]. 

The H-bonding between the variants has shifted, but the present findings show that the critical conserved interaction Asp38-Tyr449 remained consistent in Omicron. However, the interaction of more conserved H-bonds changed. These bonds were no longer detected in Asp38-Gly496 and Lys353-Gly496 in BA.1, but were changed to Asp38-Arg498 in BA.2. The replacement of salt-bridge Glu35-Arg493 from H-bond was also observed in another previous study [50]. A new salt bridge, Asp38-Arg498, was also observed. 

Additionally, no H-bond was detected in Asp38-Gly496 in BA.2. The conserved H-bond Lys353-Gly496 was found only in BA.2 and Delta, but not in BA.1. H-bonds are critical in drug–receptor interactions because they help maintain the biomolecules’ structural integrity and three-dimensional conformation [51,52]. H-bonds also play a significant role in protein–protein interactions, owing to their stronger bonds than van der Waals interactions, despite their weakness in comparison to covalent or ionic bonds [53]. On both Omicrons, the salt-bridges have been changed from Asp30-Lys417 to Glu35-Arg493 and Asp48-Arg498; on BA.2, an additional salt-bridge Glu37-His505 has been added. It is necessary to pay attention to changes in the salt bridge because it is the strongest known non-covalent molecular interaction [54]. 

The number of mutations in RBD Omicron has an impact on changes in the dynamics of its interaction with hACE2, thereby affecting the affinity between the two proteins. Given that the structure of a viral protein is critical to its function, any changes in the shape of the structure will affect the virus’ function, virulence, infectivity, and transmission rates. In fact, it is worthwhile to keep track of even the smallest changes in amino-acid composition because they may be phenotypically significant [55]. According to previous research, the mutation L452R enhances the affinity of RBD for hACE2 [56]. A significant increase in this protein’s affinity for hACE2 was observed when mutations were present in the RBD at amino acids 417, 484, and N501 [26]. Additionally, research on amino-acid–amino-acid bond pair units (AABPU) confirmed that mutation in Omicron increases the affinity of receptor-binding motif (RBM) for ACE2 [57].

The RMSD profile indicates that the hACE2-Omicron BA.2 complex was more stable compared to the other variants from 40 ns to the end of the simulation (100 nm) and fluctuated between 0.2 and 0.3 nm. The RMSD trajectories were used to make predictions about protein stability. An increase in the RMSD reflects the reduction in the stability of the protein structure [58]. The RMSF profiles indicated that BA.2 was more flexible between residues 475 and 485, although fluctuations appeared to be between 0.2 and 0.3 nm. While a lower RMSF indicates a more restricted range of movements during simulation, a higher RMSF indicates a more flexible range of movements [58]. Based on the Rg plot, hACE2-Omicron BA.2 complex showed stronger interaction compared to other variants. 

By incorporating imprecise estimates, all reported cases indicate that Omicron is, on average, far less severe than Delta [59]. Omicron replicated 70 times faster in ex vivo respiratory tract cultures than Delta in human bronchus. It was, however, 10 times slower than Delta in human lung tissues, which explains Omicron’s rapid spread while exhibiting a lower disease severity [60]. Additionally, other evidence indicated that Omicron was more likely to infect more respiratory epithelial cells, even at low-exposure doses [61]. A study also found that hamsters infected with Omicron had milder symptoms of lung infection, clinical disease, and pathology when compared to hamsters infected with previous SARS-CoV-2 variants of concern. [62].

## 5. Conclusions

Mutations in SARS-CoV-2 change the course of the epidemic day by day. Mutations in the spike protein RBD unit, where the virus attaches to the hACE2 enzyme, directly affect the rate of transmission. In this study, the 3D structure of the RBD unit of the Omicron BA.2 variant was created by a molecular modeling technique and docked to hACE2. In addition, Omicron BA.1 (PDB ID: 7WBP) and Delta (PDB ID: 7WBQ) variants, in which the structure was elucidated, were performed for redocking. According to docking interaction energies and interface-interaction analysis, Omicron BA2 exhibited a higher affinity for hACE2 than Omicron BA1. From molecular dynamics simulations, it was concluded that the strong interaction between protein–protein complexes persisted.

## Figures and Tables

**Table 1:** Mutation sites in the RBD of Omicron and Delta variants.

Variants	PANGO Lineage	Mutation Sites														
		371	373	375	376	408	417	440	446	452	477	484	493	496	498	501
Delta	B.1.617.2	S	S	S	T	R	K	N	G	R	S	E	Q	G	Q	N
Omicron BA.1	B.1.1.529	L	P	F	T	R	N	K	S	L	N	A	R	S	R	Y
Omicron BA.2	B.1.1.529	F	P	F	N	S	N	K	G	L	N	A	R	S	R	Y

**Table 2:** The HDOCK energy score, ligand RMSD (Å), binding affinity (ΔG) and dissociation constant (Kd) predicted values of Delta-hACE2, Omicron BA.1-hACE2 and Omicron BA.2-hACE2 protein–protein complex.

	Protein–Protein Complex of SARS-CoV-2 RBDs and hACE2		
Parameters	Delta-hACE2	Omicron BA.1-hACE2	Omicron BA.2-hACE2
Docking energy score	−339.88	−360.96	−369.70
Ligand RMSD (Å)	0.39	0.50	0.47
ΔG (kcal/mol)	−12.5	−11.1	−12.6
Kd (M) at 37.0 ℃	1.5 × 10^−9^	1.5 × 10^−8^	1.4 × 10^−9^

**Table 3:** The interface statistics of the interaction between the RBD and hACE2.

Variants	Number of Interface Residues		Interface Area (Å^2^)	No. of Salt Bridges	No. of Disulfide Bonds	No. of Hydrogen Bonds	No. of Non-Bonded Contacts
Omicron BA.2	hACE2	21	961	3	0	21	215
	RBD	19	1016				
Omicron BA.1	hACE2	18	847	2	0	13	120
	RBD	16	868				
Delta	hACE2	17	839	1	0	11	103
	RBD	17	885				
WT	hACE2	20	825	1	0	10	101
	RBD	17	863				

**Table 4:** The amino acid interactions between RBD of SARS-CoV-2 variant and hACE2.

hACE2	Omicron BA.2	Omicron BA.1	Delta	WT
Hydrogen bonds				
Ser19	Asn477	Asn477		
Ser19	Ala475	Ala475		
Gln24	Asn487	Asn487	Asn487	Asn487
Asp30			Lys417	Lys417
Lys31			Gln493	
His34	Arg493			
His34	Arg493			
His34	Tyr453	Tyr453		
His34	Tyr453	Tyr453		
His34		Ser494		
Glu35		Arg493		
Glu37	His505		Tyr505	
Asp38	Tyr449	Tyr449		Tyr449
Asp38		Arg498		
Tyr41	Thr500	Thr500	Thr500	Thr500
Tyr41	Thr500	Thr500	Thr500	Thr500
Tyr41	Thr500			
Gln42	Arg498			
Gln42	Gly446		Gly446	Gly446
Gln42	Tyr449		Tyr449	Tyr449
Tyr83	Asn487	Asn487	Asn487	Asn487
Tyr83	Tyr489			
Asn330	Thr500			
Lys353	Gly496		Gly496	Gly496
Lys353	Tyr501			
Lys353	Gly502	Gly502	Gly502	Gly502
Salt bridges				
Asp30			Lys417	Lys417
Glu35	Arg493	Arg493		
Glu37	His505			
Asp38	Arg498	Arg498		

## Data Availability

The authors will make the raw data supporting the conclusions of this manuscript available to any qualified researcher.

## Supplementary Materials

The following supporting information can be downloaded at: https://www.mdpi.com/article/10.3390/biology11050797/s1, Video S1: The MD simulations movie of 500 snapshots between 0 and 100 ns of SARS-CoV-2 Omicron BA.2 spike protein and hACE2; Video S2: The MD simulations movie of 500 snapshots between 0 and 100 ns of SARS-CoV-2 Omicron BA.1 RBD and hACE2; Video S3: The MD simulations movie of 500 snapshots between 0 and 100 ns of SARS-CoV-2 Delta RBD and hACE2.
