# Supplementary material for: An Insight Based on Computational Analysis of the Interaction between the Receptor-Binding Domain of the Omicron Variants and Human Angiotensin-Converting Enzyme 2
Source: Biology (Basel). 2022 May 23;11(5):797. doi: 10.3390/biology11050797 (PMC9171583; doi:10.3390/biology11050797)
Supplement: Supplementary file 1 [file biology-11-00797-s001.zip › Supplementary Material - Video/BA2 Supplementary material - VS2.pptx]

## Slide 1
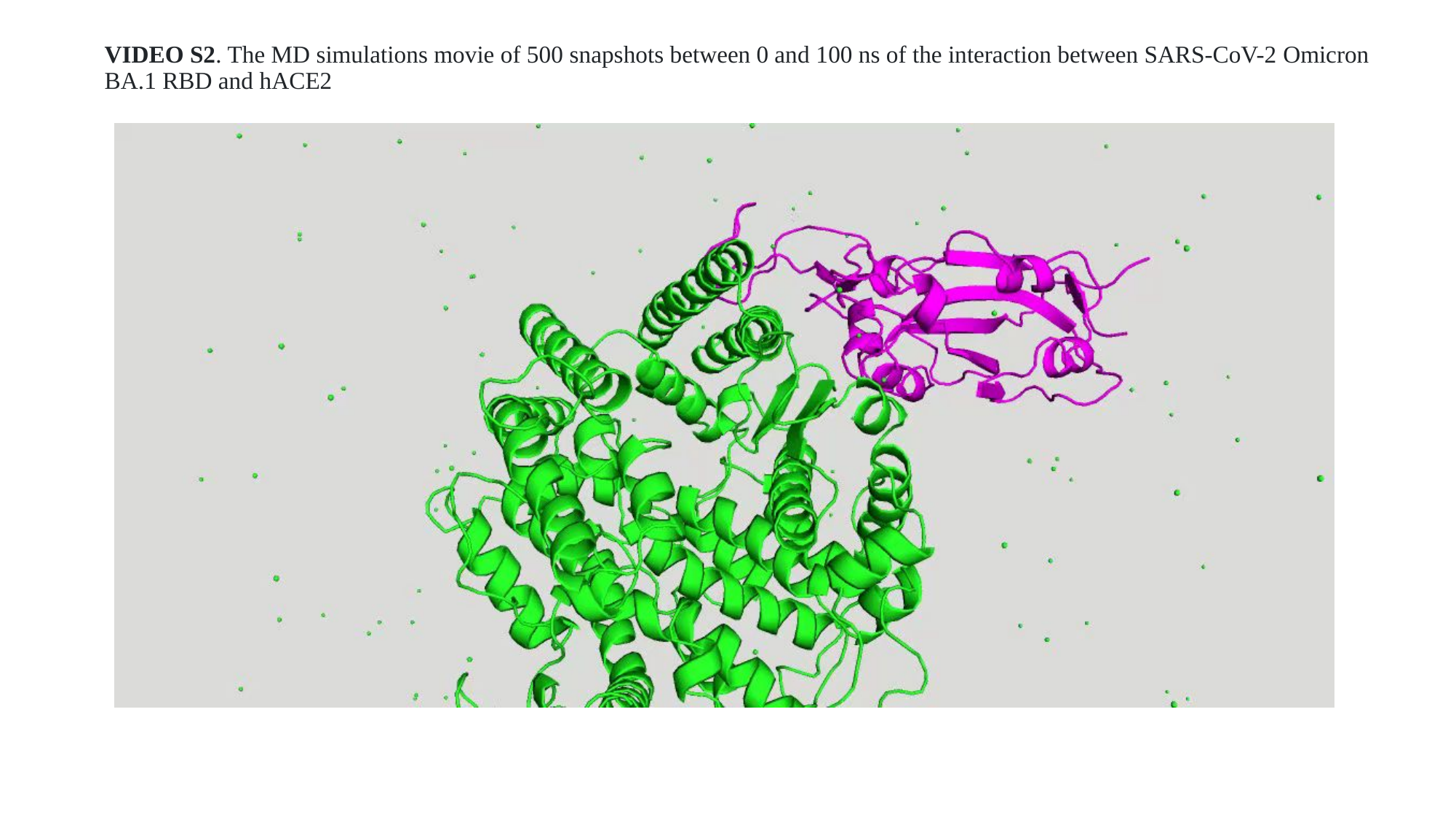

# VIDEO S2. The MD simulations movie of 500 snapshots between 0 and 100 ns of the interaction between SARS-CoV-2 Omicron BA.1 RBD and hACE2
